# Triptriolide Alleviates Lipopolysaccharide-Induced Liver Injury by Nrf2 and NF-κB Signaling Pathways

**DOI:** 10.3389/fphar.2018.00999

**Published:** 2018-08-29

**Authors:** Yi-Qi Yang, Xiao-Teng Yan, Kai Wang, Rui-Min Tian, Zhao-Yu Lu, Li-Lan Wu, Hong-Tao Xu, Yun-Shan Wu, Xu-Sheng Liu, Wei Mao, Peng Xu, Bo Liu

**Affiliations:** ^1^The Second Clinical Medical College, Guangzhou University of Chinese Medicine, Guangzhou, China; ^2^Affiliated Huai’an Hospital, Xuzhou Medical University, Huai’an, China; ^3^Guangdong Provincial Academy of Chinese Medical Sciences, Guangzhou, China; ^4^Shanghai Institute for Advanced Immunochemical Studies, ShanghaiTech University, Shanghai, China

**Keywords:** triptriolide, *Tripterygium wilfordii*, inflammation, macrophage, Nrf2

## Abstract

Nrf2 (Nuclear Factor Erythroid 2 Related Factor 2) transcription factor not only regulates oxidative stress response, but also represses inflammation by regulating cytokines production and cross-talking with NF-κB signaling pathways. Nrf2 plays an essential role in liver injury induced by oxidative stress and inflammation. Triptriolide (T11) is a minor component of *Tripterygium wilfordii* Hook F. (TwHF), which can be obtained by hydrolysis reaction of triptolide (T9). The major purpose of this study is to clarify the regulating effects of T11 on oxidative stress and inflammation *in vivo* and *in vitro*. LPS-stimulated RAW 264.7 cells were used to verify the regulating effects of T11 on oxidative stress (ROS and Nrf2 signaling pathway) and inflammatory cytokines production (TNF-α, IL-6 and IL-1β). The antioxidant responsive element (ARE) luciferase assay was employed to evaluate Nrf2 activation effect of T11 in HEK-293T cells. Lipopolysaccharides (LPS) induced acute liver injury (ALI) in BALB/c mice were used to study the protective effects (ALT, AST, MDA, SOD, histopathology and neutrophils/macrophages filtration) and the underlying protection mechanisms of ALI amelioration (Nrf2 and NF-κB signaling pathway) of T11. Firstly, the results showed that T11 can not only effectively decrease the productions of inflammatory cytokines (TNF-α, IL-6 and IL-1β), ROS and NO in LPS-stimulated RAW 264.7 cells, but also further significantly increase the activity of Nrf2 in HEK-293T cells. Secondly, the results suggested that T11 could dramatically decrease the oxidative stress responses (SOD and MDA) and inflammation (histopathology, neutrophils/macrophages filtration, TNF-α, IL-6 and IL-1β production) in LPS-induced ALI in BALB/c mice. Finally, the results implied that T11 could dramatically increase Nrf2 protein expression and decrease p-TAK1, p-IκBα and NF-κB protein expression both *in vivo* and *in vitro.* In conclusion, our findings indicated that T11 could alleviate LPS induced oxidative stress and inflammation by regulating Nrf2 and NF-κB signaling pathways *in vitro* and *in vivo*, which offers a novel insights for the application of TwHF in clinical.

## Introduction

Acute liver injury is a question of common concern for both patients and clinicians. Taking too much inflammatory and oxidative stress are the common causes of ALI (Janssenheininger et al., 2000; Koyama and Brenner, 2017). When oxidative stress occurs, oxygen free radicals and ROS accumulate in liver cells (Gustot et al., 2009). The facts would induce lipid peroxidation and membrane depolarization, along with kupffer cells (macrophages) and neutrophils infiltration, finally result in hepatocyte mitochondrial dysfunction, oxidant stress, inflammation and cell death (Haddad, 2002). There are convincing evidences show that the major cause of ALI is severe oxidative stress (Macdonald et al., 2003). Therefore, intervention with antioxidant therapy to restore the redox balance is very necessary for ALI treatment (Kamouni et al., 2017; Levada et al., 2018).

Lipopolysaccharides, an important constituent of Gram-negative bacteria cell walls, is a powerful inflammagen and can activate the immune system (Singh and Jiang, 2003). LPS-induced sepsis often leads to multi-organ failure, in particular liver injury. It mainly due to uncontrolled inflammatory cytokines secretion and obvious neutrophils infiltration in liver, and ultimately lead to inflammation and oxidative stress (Li et al., 2016). Inflammation and oxidative stress are considered as an extremely related event with the pathological process of ALI (Kolac et al., 2017; Jung et al., 2018). Under stimulation of the LPS, the classical NF-κB signaling pathway is activated to release many pro-inflammatory cytokines such as IL-1β, IL-6 and TNF-α, thereby further aggravate inflammation damage in liver (Li L. et al., 2017). In addition, LPS-induced liver injury can also bring out oxidative stress to increase the over formation of MDA, ROS and decrease the expression of SOD (Cuschieri and Maier, 2007; Lugrin et al., 2014). Recent studies implied that Nrf2 transcription factor plays an essential role in the amelioration of various inflammatory diseases (Kobayashi et al., 2016), including ALI and hepatitis (Han et al., 2010; Li et al., 2018). As an upstream regulator, Nrf2 not only regulates oxidative stress response, but also represses inflammation by regulating cytokines production (Keleku-Lukwete et al., 2017). Therefore, focusing on the inhibitory action of oxidative stress and/or inflammation may be potential strategies for the prevention and treatment of ALI (Calkins et al., 2009; Del Campo et al., 2018).

*Tripterygium wilfordii* Hook F. is a traditional Chinese herb, which has been widely used to treat autoimmune and inflammatory diseases for many years in China, such as Rheumatoid arthritis (RA), Systemic lupus erythematosus (SLE) and Glomerulonephritis (Law et al., 2011; Li et al., 2015). Currently, more than 200 compounds have been identified from the extracts of TwHF (Wan et al., 2012). Most of them show extensive pharmacological effects on anti-inflammation, immunosuppress, anti-tumor and anti-oxidant (Tao and Lipsky, 2000; Marks, 2011; Lu et al., 2014). The T11 was isolated from TwHF by Kupchan et al. (1972), and it can significantly inhibit the transcription of pro-inflammation genes in a dose dependent manner (Ma et al., 2007). However, up to now, studies about T11 have focused on its chemical structures and preparation, as well as few reports have been reported on its pharmacological effects (Liu et al., 2015). Due to the severe liver hepatotoxicity of T9, TwHF is limited in liver diseases treatment. While, we found that the T11 is a functional component of TwHF with low biological toxicity, and exerts major detoxification effect in TwHF (**Supplementary Table S1**). Therefore, it would be necessary to conduct a comprehensive anti-inflammation and anti-oxidant studies of T11, and clarify the inner mechanisms in it.

In the present study, we investigated the protective effect of T11 on LPS-induced oxidative stress and inflammation *in vivo* and *in vitro*, and further explored the underlying mechanism in it. Our results not only provide meaningful information for the development and use of T11, but also offer novel insights into the mechanisms of TwHF in inflammatory diseases.

## Materials and Methods

### Reagents and Chemicals

Triptriolide, purity > 98 %, was obtained from Liubo Laboratory (GuangZhou, China). LPS (*Escherichia coli* 055:B5) was purchased from Sigma-Aldrich (St. Louis, MO, United States). ALT, AST, MDA, and SOD test kits were obtained from Nanjing Jiancheng Bioengineering Institute (Nanjing, China). Mouse TNF-α, IL-1β, and IL-6 ELISA kits were purchased from eBioscience (San Diego, CA, United States). ROS test kit and NO test kit were purchased from Beyotime Biotechnology (Shanghai, China). Antibodies against TAK1 (#5206), p-TAK1 (#9339), IκBα (#4814), p-IκBα (#2859), β-Actin (#3700), NF-κB(p65) (#8242), Nrf2(#12721), Keap1(#8047),and GAPDH (#5174) were purchased from Cell Signaling Technology Inc. (Beverly, MA, United States). Antibodies against TAB1(ab76412), CD68 (ab125212) antibodies and Goat Anti-Rat IgG H&L (Alexa Fluor^®^ 488) antibody (ab150157) were purchased from abcam (Abcam, Cambridge, MS, United States). Ly6G (sc-53515) was obtained from Santa (Santa, CA, United States). Alexa Fluor 488 Donkey anti-Rabbit IgG (H+L) Highly Cross-Adsorbed Secondary Antibody (#A-21206) was purchased from Invitrogen (Thermal Scientific). Fetal bovine serum (FBS), RPMI Medium 1640, Dulbecco’s modified Eagle’s medium (DMEM) and phosphate buffer saline (PBS) were purchased from GIBCO Laboratories (Grand Island, NY, United States). Dimethyl Sulphoxide (DMSO), 4′,6-Diamidino-2-phenylindole (DAPI), Penicillin, streptomycin, MTT and Sodium carboxymethyl cellulose (CMC) were purchased from Sigma-Aldrich (St. Louis, MO, United States). Plastic materials were purchased from Falcon Labware (Becton-Dickinson, Franklin Lakes, NJ, United States).

### Preparation and Identification of T11

The preparation method of T11 was performed as described by Musser (2000), with slight modification. Briefly, T9 (456 mg, 1.27 mmol) was suspended in phosphate buffer (pH 4.0, 250 mL) at room temperature. Then the reaction mixture was heated to reflux in the oil bath at 120°C for 48 h. After the solvent was removed by rotary evaporation, the residual solid was re-dissolved in methanol, and centrifuged at 12000 rpm for 5 min at room temperature. The supernatant was filtered through a 0.22 μm membrane filter. The crude product was isolated and purified by flash column chromatography to gain final product (236 mg, 0.62 mmol, 49.2% yield). The purity of the product was detected as following condition: the sample was diluted with methanol and passed through the 0.22 μm membrane filter, and analyzed by UPLC system with BEH Shield RP18 column (2.1 × 100 mm, 1.7 μm) at 35°C. Acetonitrile and Q-water were used as mobile phases, flow rate was 0.3 mL/min, the UPLC gradient was showed in **Table 1**. The chemical structure of T11 was confirmed by ^1^H-NMR, ^13^C-NMR and HRMS (High resolution mass spectra).

**Table 1:** The UPLC gradient and mobile phases: Acetonitrile and Q-water.

Time (min)	0	4	7	12	16	17	18
Acetonitrile (%)	15	18	20	28	35	15	15
Water (%)	85	82	80	72	65	85	85


### Animals and Treatment

Male BALB/c mice (18–20 g) were used for this experiment. The BALB/c mice were purchased from Laboratory Animal Services Center, Guangzhou University of Chinese Medicine (Guangzhou, China). After arrival in our facility, the National Institutes of Health Guide for the use of Laboratory animals were performed to raise all animals. Animals were raised in a standard environment condition with the temperature at 22 ± 2°C under a 12 h dark/light cycle, and allowed free access to sterilized water and standard food. Experimental protocols were reviewed and approved by Animal Care and Use Committee (Guangzhou University of Chinese Medicine, Guangzhou).

Acute liver injury mice model was induced as described by Wang et al. (2018). Briefly, mice were randomly divided into 6 groups (*n* = 6 per group): (1) Normal (0.4% CMC), (2) Model (3 mg/kg LPS), (3) LPS+T11-L (2.8 mg/kg, Low dose), (4) LPS+T11-M (14 mg/kg, Middle dose), (5) LPS+T11-H (28 mg/kg, High dose), (6) LPS+PA (Prednisone acetate, PA as a positive control, 1.3 mg/kg). T11 and PA were pretreated by gavage for 7 days. LPS was administered through intraperitoneal injection at 6 h before sacrificed. Then serum samples and liver tissues were isolated for downstream biochemistry and molecular analysis.

### RAW 264.7 Cell Culture and MTT Assay

RAW 264.7 macrophage cell lines were purchased from iCell Bioscience Inc. (Shanghai, China). RAW 264.7 macrophage cell lines were cultured in Roswell Park Memorial Institute (RPMI) 1640 medium with 10% FBS, 100 μg/mL streptomycin and 100 U/mL penicillin and then maintained in a humidified incubator at 37°C with 5% CO_2_ (Xu et al., 2017). The medium was replaced every 2 days, consecutively.

Cell viability was tested by MTT method (Liang et al., 2017a). Firstly, 100 μL RAW 264.7 macrophage cells (1 × 10^4^ cells/mL) were cultured in a 96-well plate and treated with 100 μL different dose of T11 (final concentration 0.1, 0.3, 0.4, 0.5 mM) for 24 h. Under this same condition, the medium without samples were used as the control wells, and the blank wells were medium without cells. Afterward, 20 μL of MTT (5 mg/mL) was added into each well, incubated for an additional 4 h. Then the supernatant was removed and 150 μL DMSO was added into each well. The plates were measured at 490 nm using a Multiskan Go. The cell viability (%) was calculated as formula (1):

$$\text{Cell viability}(\%)=\frac{As-Ab}{Ac-Ab}\times 100\%$$

where *As*, *Ac*, and *Ab* stand for the absorbance values of the sample wells with different T11 concentrations wells, control wells and blank wells, respectively.

### Measurement of Cytokines and NO in RAW 264.7 Cell

RAW264.7 cells (2 × 10^6^ cells/mL) were seeded into 6-well plates. The cells were treated with different concentrations of T11 (final concentration 0.1, 0.3, 0.4, 0.5 mM) in the presence of LPS (1 μg/mL) stimulation. The level of NO in cell supernatant was detected using NO test kit (Xiang et al., 2013), the levels of cytokines (TNF-α, IL-1β, and IL-6) were determined by commercially available enzyme linked immunosorbent assay (ELISA) kits (Lou et al., 2015). The detection methods were performed according to the manufactures’ instruction.

### Flow Cytometry Assay and Intracellular ROS Measurement

The ROS assay kit is a most commonly used method for quantitative detection of ROS in cells (Zhang et al., 2014), which is based on fluorescence intensity change of fluorescent dye DCFH-DA (2,7-Dichlorodi -hydrofluorescein diacetate). While, DCFH-DA itself has no fluorescence and could cross the cell membrane freely. It can be hydrolyzed by the intracellular esterase to produce DCFH, DCFH can not permeate the cell membrane, so the probe is easy to load into the cell. The ROS in the cell can oxidize the non-fluorescent DCFH into fluorescent DCF. The fluorescence intensity is proportional to the level of ROS, the real level of ROS can be analyzed by flow cytometry (BD Calibur) and a multi-detection reader (Tecan Infinite M1000Pro) at the maximum excitation wavelength of 480 nm and the maximum emission wavelength of 525 nm, respectively.

RAW264.7 cells (2 × 10^6^ cells/mL) were seeded into 6-well plates. The cells were treated with different concentrations of T11 (0.1, 0.3, 0.4, 0.5 mM) in the presence of LPS (1 μg/mL) stimulation for 24 h, then the cells were washed twice with PBS and stained with DCFH-DA (1:1000 dilution with FBS free RPMI 1640 medium) in a humidified incubator at 37°C with 5% CO_2_ for 30 min, finally the fluorescence intensities were detected by flow cytometry and a multi-detection reader, respectively. All samples were run in triplicate for each experiment.

### Dual Luciferase Reporter Gene Assays

Dual luciferase reporter gene assays were preformed according to Zeng et al. (2017), with slight modification. HEK 293T is a very common tool cell lines with high transfection efficiency for the expression of foreign genes, which was obtained from Chen Laboratory (Guangzhou, China) (Chen et al., 2016). HEK 293T cells were cultured in DMEM medium with 10 % FBS, 100 μg/mL streptomycin and 100 U/mL penicillin and then maintained in a humidified incubator at 37°C with 5% CO_2_. The medium was replaced every 2 days, consecutively. For the dual luciferase reporter gene assay, HEK 293T cells (2 × 10^6^ cells/mL) were seeded into 6-well plates. When cell density reached about 75% confluence, the antioxidant responsive element (ARE)-luciferase plasmid pGL4.22 (luc2CP/Puro) and Renilla luciferase expression plasmid pGL4.74 (hRluc/TK) were cotransfected into cells using Lipo6000^TM^ Transfection Reagent in accordance with the manufacturer’s protocol (Beyotime Biotechnology, Shanghai, China). After 18 h transfected, the cells were treated with different concentrations of T11 (0.1, 0.3, 0.4, 0.5 mM) for 24 h, and both firefly and Renilla luciferase activities were measured with the dual Luciferase Reporter Gene Assay Kit (Beyotime Biotechnology, Shanghai, China). The experiment was carried out in triplicate for each test.

### Evaluation of Liver Coefficient

All mice were weighed every day before sacrificed, the food and water intake were recoded daily. Liver tissues were collected and weighed immediately after 6 h of LPS injection, the liver coefficient was measured by a formula (Yong et al., 2018) as follows (2):

$$\text{The liver coefficient}(\%)=\frac{Ws}{Wb}\times 100\%$$

where *Ws* and *Wb* were the organ weight and body weight, respectively.

### Measurement of Liver Function and Oxidative Stress in Serum and Liver Tissues

All mice were killed by anesthesia with an overdose of 10% chloral hydrate, the serum samples and liver tissues were collected immediately after 6 h of LPS injection. The blood samples were drawn from orbit and transferred to 2 mL EP tubes, then centrifugated at 3000 rpm for 15 min to obtain serum samples (Liang et al., 2017b). The liver tissues were homogenized in PBS, and further centrifugated at 12000 rpm for 15 min to obtain cell lysate samples. The levels of ALT, AST, MDA and SOD in serum and liver samples were assayed using commercial test kits. All methods were performed according to the manufacturer’s instructions, respectively (Wei et al., 2014; Dong et al., 2015). Each experiment was performed in triplicated.

### Evaluation of Liver Histopathology

The changes of liver histopathology were evaluate according to report method (Hassan et al., 2017), with slight modification. Briefly, tissue samples were fixed in 4% polyoxymethylene for 24–48 h, then embedded with paraffin for long time storage. The liver tissue section was cut into 3 μm and stained by hematoxylin and eosin (H&E) to evaluate the pathological process of liver. Then the paraffin of section was removed with xylen, and tissues were rehydrated with gradient ethanol. The sections were stained in hematoxylin for 10 min and eosin for 5 min according to manufacturer’s procedures, finally dehydrated with gradient ethanol and xylene according to standard procedures. Images were captured and analyzed by light microscope (Olympus BX53, Japan). The inflammation score of liver was evaluated as described by Ishak et al. (1995). The numbers of inflammatory cells were calculated by Image J software.

### Immunohistochemistry and Immunofluorescence Assay

The methods of IHC or IF was performed as described by Moriya et al. (2012). The liver tissue section was cut into 3 μm for IHC and IF staining. Its dewaxing and rehydration processes were same as H&E method. And subsequently incubated for 30 min at room temperature in 1% Triton-X 100 solution, follow by antigen retrieval with citric acid buffer (pH 6.0) in microwave. The sections were washed twice with PBS-T and blocked using a blocking solution containing 3% bovine serum albumin for 30 min at room temperature. The primary antibody was incubated at 37°C for 2 h in a moist chamber for neutrophils (Ly6G, 1/200 dilution), macrophages (CD68, 1/200 dilution) and Nrf2 (1/200 dilution) protein staining. After thoroughly washed with PBS-T, the secondary antibody solution was added to sections and incubated at 37°C in a moist chamber for 1 h. Alexa Fluor 488 marked secondary antibodies (1/500 dilution) and horseradish peroxidase-conjugated secondary antibodies (HRP, 1/500 dilution) were used in IF and IHC staining, respectively. DAPI staining was applied for nuclear staining in IF. Images were captured by using confocal fluorescence microscope (Zeiss LSM710, Germany) or microscope (Olympus BX53, Japan). The intensity of IF pictures was calculated using ZEM software. In addition, the positive expression of IHC was analyzed by Image J software.

### Measurement of Cytokines in Liver

The liver tissues were homogenized in PBS, and centrifuged at 12000 rpm for 15 min, collected supernatants for the measurement of cytokines (TNF-α, IL-1β, and IL-6) by ELISA kits (Chen et al., 2017), the detection methods were performed according to the manufactures’ instruction.

### Western Blotting

The method of Western Blotting was performed according to Liang et al. (2017a), with minor modifications. In brief, total proteins were isolated in protein lysate buffer with proteinase inhibitor phenylmethanesulfonyl fluoride (PMSF) and phosphatase inhibitors (PhosSTOP, Roche). The collected solution was centrifuged at 12000 rpm for 15 min, the precipitate was discarded, and loading buffer was added into supernatant, then the mixture was boiled for 10 min. The equal amount of proteins from each group were loaded on to 12% SDS-polyacrylamide gel electrophoresis (SDS-PAGE) gel, with low voltage at 100 V and high voltage at 120 V to separate the target protein. Then the target protein band was transferred onto nitrocellulose membrane. The membrane was blocked with 5% non-fat milk for 2 h at room temperature to decrease the background of secondary antibody, then incubated with primary antibodies (including, TAK1, p-TAK1, TAB1, IκBα, p-IκBα, NF-κB, Nrf2, keap1, GAPGH and β-actin) solution overnight at 4°C. After thoroughly washed with Tris buffered saline with Tween-20 (TBS-T), the Horseradish Peroxidase (HRP)-conjugated secondary antibody solution was added to membrane and incubated at 37°C in a moist chamber for 1 h. After thoroughly washed with TBS-T, ECL substrate was added to nitrocellulose membrane in a dark condition. Then protein blots were immediately detected by Las 4000 imager. The intensity of each lane was quantified by Image J Lab software.

### Statistical Analysis

Statistical analysis was performed with SPSS version 18.0 (Chicago, IL, United States). All results were expressed as mean ± SEM. Group comparisons were performed by one-way analysis of variance (ANOVA) followed by *post hoc* Tukey’s test or Student’s *t*-test when appropriate. *P* < 0.05 was considered as statistically significant.

## Results

### The Purity and Structure of T11

**FIGURE 1:**
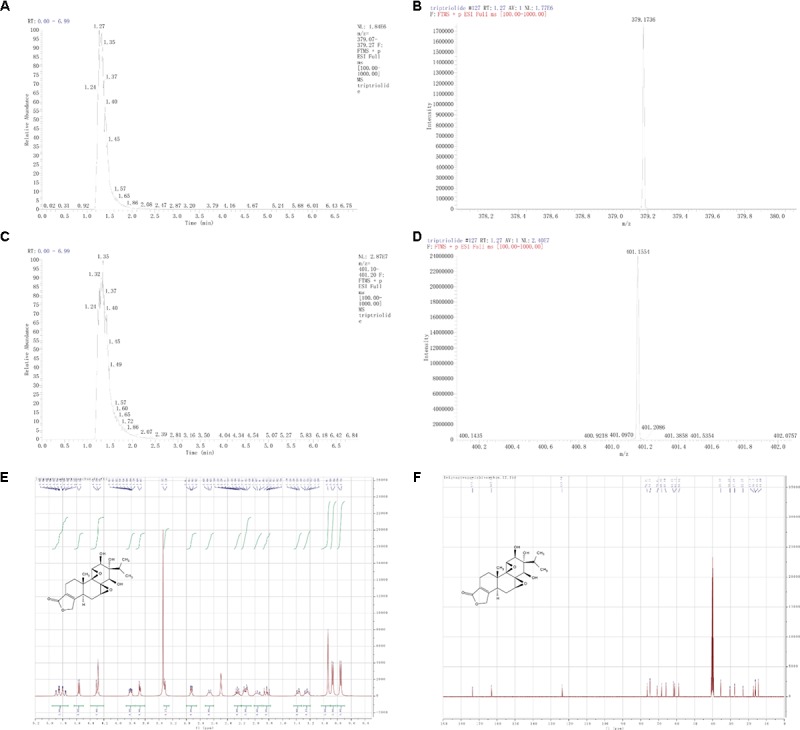
Structure characterization of T11. **(A)** [M+H]^+^ extracted ion chromatogram (EIC) of high resolution mass spectra (HRMS). **(B)** Mass peak of [M+H]^+^. **(C)** [M+Na]^+^ EIC of HRMS. **(D)** Mass peak of [M+Na]^+^. **(E)** ^1^H-NMR. **(F)** ^13^C-NMR.

The purity of T11 was ≥98% as detected by UPLC method. The structure of T11 and its high resolution mass spectra, spectrum of ^1^H and ^13^C-NMR were shown in **Figure 1**. T11: white powder, HRMS-ESI calcd. For C_20_H_26_O_7_: [M+H]^+^ 379.1757, found 379.1735; [M+Na]^+^ 401.1576, found 401.1553. ^1^H-NMR (400 MHz, DMSO-*d*6): δ 4.96 – 4.72 (m, 2H), 4.56 (d, *J* = 7.4 Hz, 1H), 4.39 – 4.20 (m, 2H), 3.88 – 3.75 (m, 1H), 3.68 (d, *J* = 5.4 Hz, 1H), 3.31 (d, *J* = 6.0 Hz, 1H), 2.93 (dd, *J* = 7.4, 1.9 Hz, 1H), 2.66 (s, 1H), 2.26 (q, *J* = 6.9 Hz, 1H), 2.14 (dt, *J* = 14.9, 5.7 Hz, 2H), 1.97 (s, 1H), 1.89 – 1.77 (m, 1H), 1.37 (dd, *J* = 12.6, 5.3 Hz, 1H), 1.26 (dd, *J* = 12.1, 6.0 Hz, 1H), 0.94 (s, 3H), 0.87 (d, *J* = 6.8 Hz, 3H), 0.76 (d, *J* = 6.9 Hz, 3H).^13^C-NMR (101 MHz, DMSO-*d*6): δ 173.71, 163.03, 123.66, 76.31, 74.77, 70.81, 68.30, 65.84, 61.60, 61.10, 58.82, 35.33, 30.25, 27.68, 22.95, 17.16, 16.17, 15.99, 14.44.

### T11 Exposure Reduced the Productions of Inflammatory Cytokines, ROS and NO in LPS-Stimulated RAW 264.7 Cells

**FIGURE 2:**
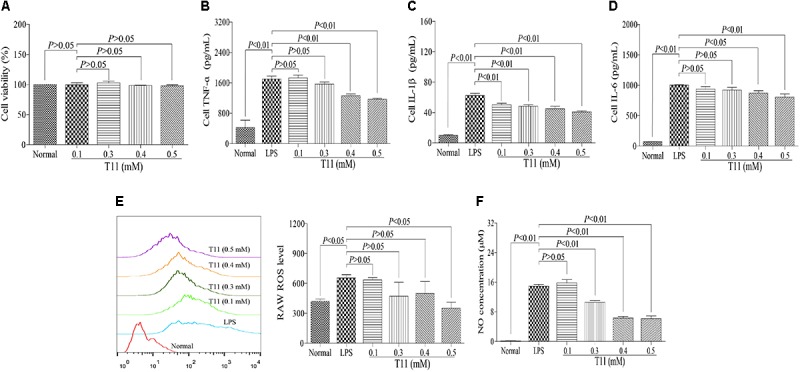
The effects of T11 on the secretion levels of pro-inflammatory cytokines and inflammatory mediators in LPS-stimulated RAW 264.7 cells. **(A)** Cell viability. **(B)** The level of TNF-α. **(C)** The level of IL-1β. **(D)** The level of IL-6. **(E)** The level of ROS. **(F)** The level of NO. The data reveal as the mean ± SEM (*n* = 3 in each group). Group comparisons were performed by one-way analysis of variance (ANOVA) followed by *post hoc* Tukey’s test or Student’s *t*-test when appropriate. *P* < 0.05 was considered as statistical significance.

The cytotoxic effect of T11 was evaluated by MTT assay. The result showed that 0.1, 0.3, 0.4, and 0.5 mM T11 did not cause the cytotoxic effect on RAW264.7 cells (**Figure 2A**). And then, we studied whether T11 could reduce cytokines (TNF-α, IL-6, IL-1β) production in LPS-stimulated RAW 264.7 cells. As shown in **Figures 2B–D**, the production of TNF-α, IL-6, IL-1β were significantly increased in LPS group compared to those of normal group. And the T11 treatment could dramatically inhibit the productions of TNF-α, IL-6, and IL-1β in a dose dependant manner, when compared with LPS group. In addition, we further investigated whether T11 could reduce the levels of ROS and NO in LPS-stimulated RAW 264.7 cells. And under the stimulation of LPS, the productions of ROS and NO were significantly increased in RAW 264.7 cells (**Figures 2E,F**). However, the T11 treatment could significantly inhibit the productions of ROS and NO in a dose dependant manner in LPS-stimulated RAW 264.7 cells (**Figures 2E,F**).

### T11 Exposure Regulated the NF-κB Signaling Pathways in RAW 264.7 Cells

**FIGURE 3:**
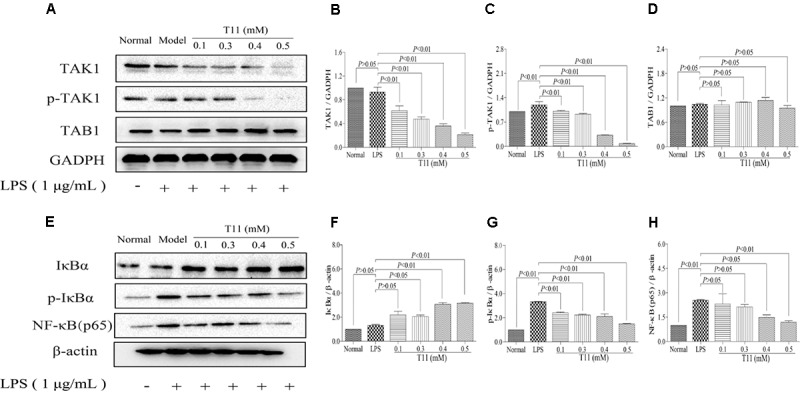
T11 decreased the activation of NF-κB signaling pathway in LPS-stimulated RAW 264.7 cells. **(A)** The proteins levels of TAK1, p-TAK1 and TAB1 were detected by western blot. **(B)** TAK1 expression. **(C)** p-TAK1 expression. **(D)** TAB1 expression. **(E)** The proteins levels of IκBα, p-IκBα and NF-κB (p65) were detected by western blot. **(F)** IκBα expression. **(G)** p-IκBα expression. **(H)** NF-κB (p65) expression. The data reveal as the mean ± SEM (*n* = 3 in each group). Group comparisons were performed by one-way analysis of variance (ANOVA) followed by *post hoc* Tukey’s test or Student’s *t*-test when appropriate. *P* < 0.05 was considered as statistical significance.

To further investigate the inner mechanisms of T11 on LPS-stimulated RAW 264.7 cells, we detected the changes of NF-κB related signaling pathways, which are closely related to the secretion of pro-inflammatory cytokines (Tian et al., 2017). As shown in **Figure 3**, the stimulation of LPS could slightly down-regulate the expression of TAK1 and significantly up-regulate the expression of p-TAK1 without affecting the total expression of TAB1 compared to normal group. Followed by decreased IκBα and increased p-IκBα and NF-κB (p65) in LPS group compared to normal group (**Figures 3A–D**). The results of molecular docking showed that T11 had a good binding affinity for TAK1-TAB1 protein. A carbonyl group of T11 formed hydrogen bonds with Val 42, Ser 111, Leu 163 of TAK1-TAB1 protein (**Supplementary Figure S5**). Further studies revealed that T11 treatment could not only down-regulate the expressions of TAK1 and p-TAK1 in a dose independent manner in LPS-stimulated RAW 264.7 cells (**Figures 3E–H**), but also significantly down-regulate the expressions of p-IκBα and NF-κB (p65).

### T11 Adjusted Nrf2 Signaling Pathway in RAW 264.7 Cells

**FIGURE 4:**
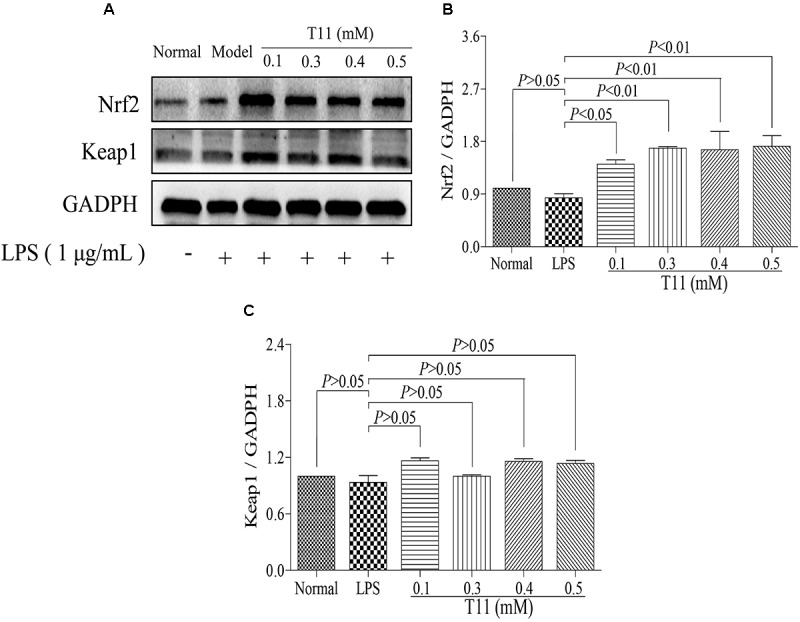
T11 could regulate Nrf2 signaling pathway in LPS-stimulated RAW264.7 cells. **(A)** The proteins levels of Nrf2 and keap1 were detected by western blot. **(B)** Nrf2 expression. **(C)** keap1 expression. The data reveal as the mean ± SEM (*n* = 3 in each group). Group comparisons were performed by one-way analysis of variance (ANOVA) followed by *post hoc* Tukey’s test or Student’s *t*-test when appropriate. *P* < 0.05 was considered as statistical significance.

Nrf2 signaling pathway plays an important role in regulations of oxidative stress and inflammation (Keleku-Lukwete et al., 2017). So, we have studied the effects of T11 on the expression of Nrf2 signal pathway in RAW 264.7 cells. Firstly, the results of molecular docking showed that T11 had good binding affinity for keap1 protein, and a carbonyl group of T11 formed hydrogen bonds with Agr483, Ser 555, Tyr 572 and Ser602 of Keap1 protein (**Supplementary Figure S6**). T11 treatment could dose-dependently up-regulate the expression of Nrf2 without affecting the expression of keap1 in RAW 264.7 cells compared to LPS group. However, the expressions of Nrf2 and keap1 in LPS group have no change compared to normal group (**Figure 4**).

### T11 Can Significantly Increase ARE Promoter Activity in HEK 293T Cells

**FIGURE 5:**
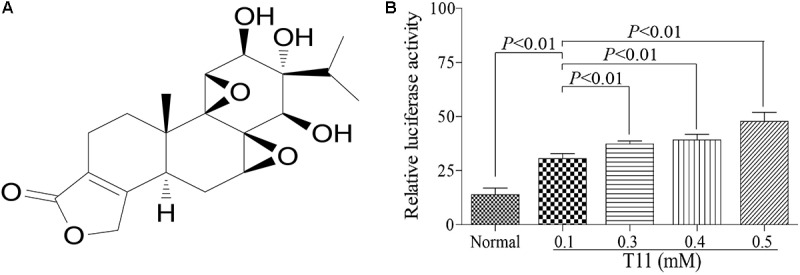
Antioxidant responsive element promoter activity identification of T11. **(A)** The chemical structure of T11. **(B)** The relative luciferase activity of T11 in HEK 293T cell. The data reveal as the mean ± SEM (*n* = 3 in each group). Group comparisons were performed by one-way analysis of variance (ANOVA) followed by *post hoc* Tukey’s test or Student’s *t*-test when appropriate. *P* < 0.05 was considered as statistical significance.

To further study the Nrf2 activation effect of T11, we used a dual luciferase reporter gene assay to evaluate the promoter efficiency of Nrf2 in HEK 293T cells with different dose of T11 (0.1, 0.3, 0.4, 0.5 mM). The reporter gene assay included a renilla luciferase gene and a firefly luciferase gene. The renilla luciferase was used as an internal control to normalize data in the experiment. As shown in **Figure 5**, T11 could significantly induce the transcription of ARE-dependent luciferase gene in a dose-dependent manner in cotransfected HEK 293T cells.

### T11 Pre-treatment Could Restore the Signs in LPS-Induced Liver Injury in Mice

**FIGURE 6:**
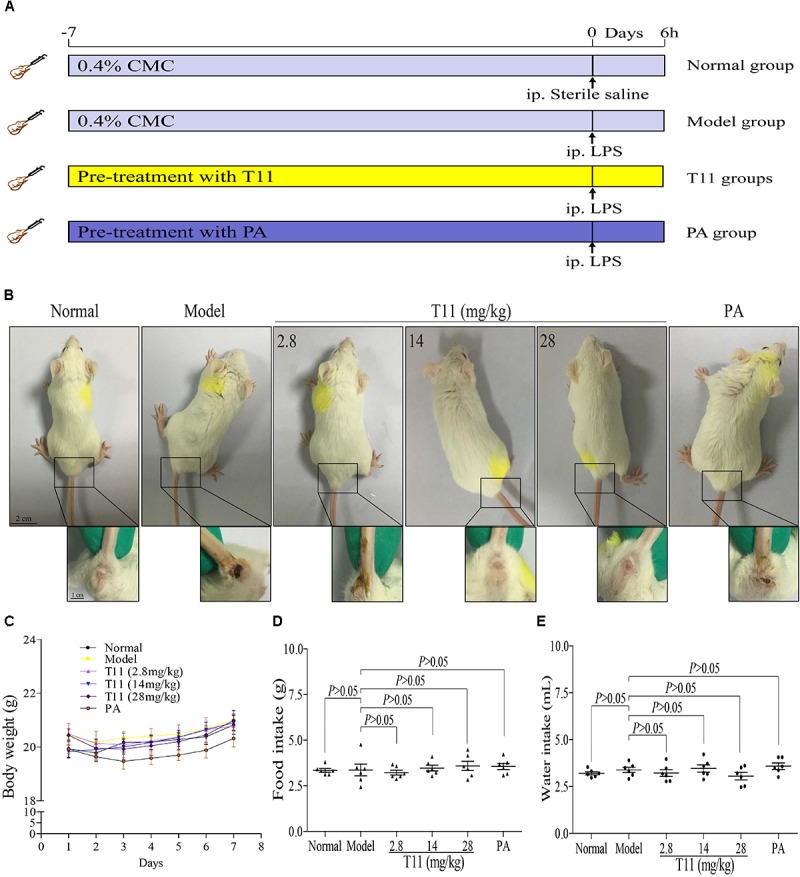
The effect of T11 on signs for LPS-induced liver injury in mice. **(A)** The schematic diagram of the experiment. **(B)** Representative sign pictures of mice in each group. **(C)** Body weight. **(D)** Food intake. **(E)** Water intake. The data revealed as the mean ± SEM of 5–7 mice in each group. Group comparisons were performed by one-way analysis of variance (ANOVA) followed by *post hoc* Tukey’s test or Student’s *t*-test when appropriate. *P* < 0.05 was considered as statistical significance.

The experiment was conducted as described in the animal treatment section (**Figure 6A**). According to the observations of animal signs and symptoms, T11 pre-treatment could effectively alleviate the special symptoms in LPS-induced mice, including depression (Adebesin et al., 2017; Li M. et al., 2017), curled up, eye secretions increase (**Supplementary Figure S3**) and severe diarrhea (**Figure 6B**). The body weight, food intake and water intake were also recorded every day. As shown in **Figures 6C–E**, The body weight, food intake and water intake had no significant difference in the seventh day of T11 and PA administration. However, the signs change showed that T11 could effectively protect the body from LPS induced injury in mice.

### T11 Pre-treatment Could Relieve Liver Injury in LPS-Induced Mice

**FIGURE 7:**
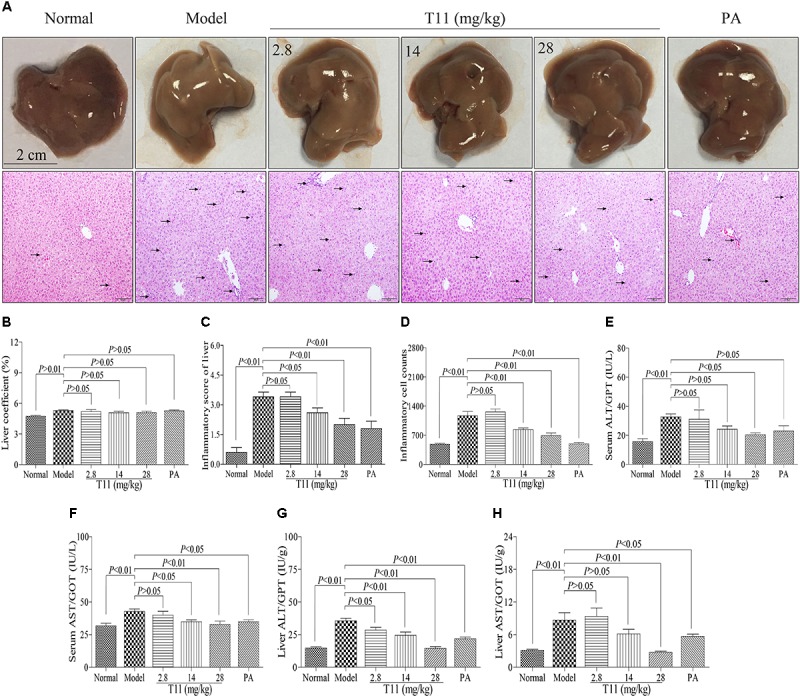
T11 relieved liver injury in LPS-induced mice. **(A)** Representative liver pictures of mice in each group (black arrows indicating inflammatory cell accumulation in H&E pictures of liver). **(B)** The inflammatory score of liver in each group. **(C)** The liver coefficient in each group. **(D)** The inflammatory cell counts in liver. **(E)** The level of ALT in serum. **(F)** The level of AST in serum. **(G)** The level of ALT in liver. **(H)** The level of AST in liver. The data reveal as the mean ± SEM of 5–7 mice in each group. Group comparisons were performed by one-way analysis of variance (ANOVA) followed by *post hoc* Tukey’s test or Student’s *t*-test when appropriate. *P* < 0.05 was considered as statistical significance.

Mice injected with LPS have characteristics of hepatic inflammation (Zhang et al., 2017). In order to evaluate the protective effect of T11 on LPS-induced liver injury, macroscopic appearance, H&E staining and liver biochemical indexes were used to analyze the changes of liver in ALI model. In normal group, the macroscopic appearance of liver revealed normal color and did not appear hepatocyte enlargement. Moreover, the histomorphology of liver showed integral hepatic architecture and radiated hepatic cord (**Figure 7A**). However, the liver macroscopic appearance of model group had changed a little white and hepatocyte enlargement. The H&E staining showed damaged hepatic architecture and inflammatory cells infiltration, as well as the inflammatory score of liver significantly increased in model group compared to normal group (**Figures 7A–D**). We also found that injection with LPS in mice for 6 h can not cause obvious collagen fibers production in liver as compared to normal group (**Supplementary Figure S4**). The animals from pre-treated with T11 and PA not only obviously improved the macroscopic appearance of liver, but also protected hepatic architecture with radiated hepatic cord and reduce inflammatory cells infiltration (**Figures 7A–D**). Meanwhile, the inflammatory score of liver significantly reduced after pre-treatment with T11 and PA as compared to model group (**Figures 7C**). In addition, the activities of serum and liver ALT and AST were significantly increased in model group compared to normal group (**Figures 7F–H**). T11 and PA pre-treatment could obviously inhibit the activities of ALT and AST in serum and liver compared to the model group (**Figures 7E–H**). Moreover, treatment T11 without LPS injection showed that T11 did not cause the liver injury in mice (**Supplementary Figures S1**, **S2**). These results implied that T11 have no toxic effect on liver and could protect liver form injury in LPS-induced ALI mice.

### T11 Pre-treatment Reduced Inflammatory Cells Infiltration and Cytokines Production in LPS-induced Liver Injury in Mice

**FIGURE 8:**
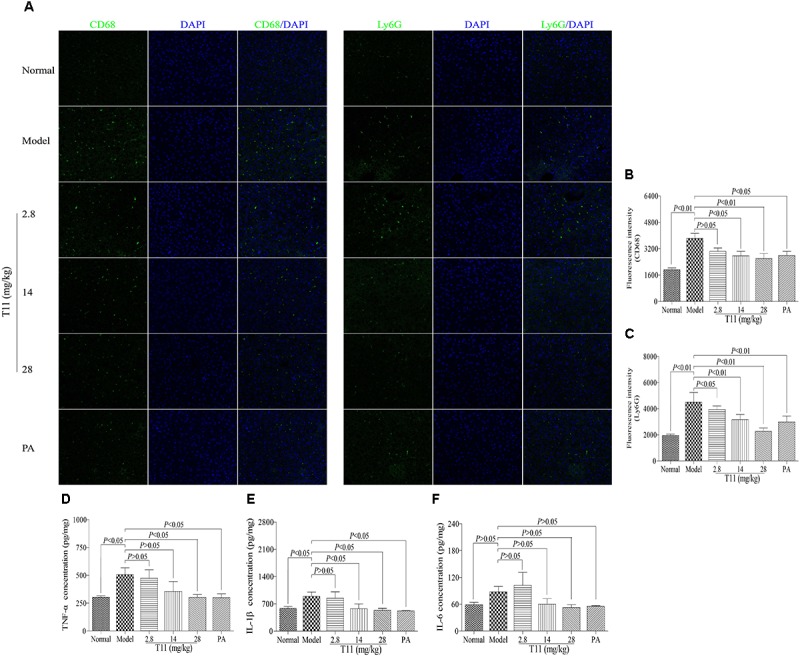
T11 alleviated inflammation in LPS-induced liver injury mice. **(A)** Immunofluorescence staining of CD68 and Ly6G in liver. **(B)** The fluorescence intensity of CD68. **(C)** The fluorescence intensity of Ly6G. **(D)** The level of TNF-α in liver. **(E)** The level of IL-1β in liver. **(F)** The level of IL-6 in liver. The data reveal as the mean ± SEM of 5–7 mice in each group. Group comparisons were performed by one-way analysis of variance (ANOVA) followed by *post hoc* Tukey’s test or Student’s *t*-test when appropriate. *P* < 0.05 was considered as statistical significance.

The LPS-induced liver injury will lead to a large number of inflammatory cells infiltration, including macrophages and neutrophils, which are the major population cells responsible for cytokines production (Tanaka et al., 2014). In order to investigate whether T11 could reduce the accumulations of macrophages and neutrophils, as well as inhibit cytokines production, IF and ELISA methods were used to assess the number of macrophages and neutrophil in liver, respectively. As shown in **Figures 8A–C**, the expressions of CD68^+^ macrophage and Ly6G^+^ neutrophil were significantly increased compared to normal group. Meanwhile, the levels of inflammatory cytokines (TNF-α and IL-1β) were also significantly elevated in model group (**Figures 8D,E**). However, pre-treatment with T11 and PA could markedly reduce the accumulations of macrophages and neutrophils in liver (**Figures 8A–C**). In addition, the serum level of TNF-α was significantly inhibited by the highest dose of T11 (28 mg/kg), while the level of IL-1β was reduced significantly in both T11-M (14 mg/kg) and T11-H (28 mg/kg) groups (**Figures 8D,E**). However, The level of IL-6 showed an upward trend in model group, but without significant difference when compared to normal group. Meanwhile, per-treatment T11 and PA could reduce the release of IL-6, but there was no significant difference compared to model group (**Figure 8F**). The reason for this might be, the time point of sample collection at 6 h after injection LPS is critical for the peak in TNF-α and IL-1β in the experiment, the peak time of IL-6 might be longer than that of TNF-α (Agelaki et al., 2002). Therefore, in the present study, T11 has a similar anti-inflammatory effect as compared to PA group. These results showed that T11 could alleviate the LPS-induced liver injury by reducing the infiltration of neutrophils and macrophages, as well as the production of cytokines (TNF-α, IL-6, and IL-1β).

### T11 Pre-treatment Alleviated LPS-stimulated Oxidative Stress in Mice

**FIGURE 9:**
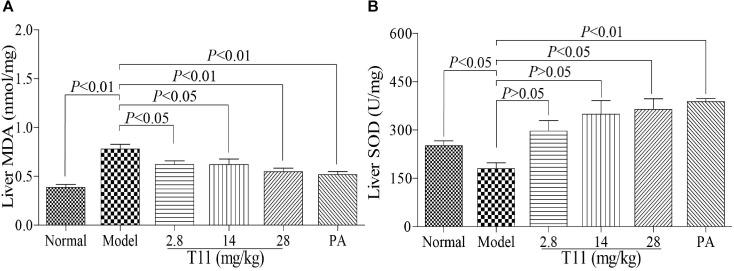
T11 alleviated oxidative stress in LPS-induced liver injury mice. **(A)** The level of MDA in liver. **(B)** The level of SOD in liver. The data reveal as the mean ± SEM of 5–7 mice in each group. Group comparisons were performed by one-way analysis of variance (ANOVA) followed by *post hoc* Tukey’s test or Student’s *t*-test when appropriate. *P* < 0.05 was considered as statistical significance.

Oxidative damage also plays an important role in LPS-induced live injury in mice (Kamouni et al., 2017). Therefore, we examined whether T11 pre-treatment could alleviate LPS-stimulated oxidative stress. As shown in **Figure 9**, the level of MDA in model group was increased significantly compared to normal group. Meanwhile, the level of SOD was markedly decreased in model group, but significantly increased in T11 groups. Therefore, the results suggested that T11 pretreatment could effectively reduce the LPS-induced MDA formation and increase the SOD generation (**Figures 9A,B**) in mice through anti-oxidative properties.

### T11 Pre-treatment Suppressed LPS-activated NF-κB Signaling Pathway in Mice

**FIGURE 10:**
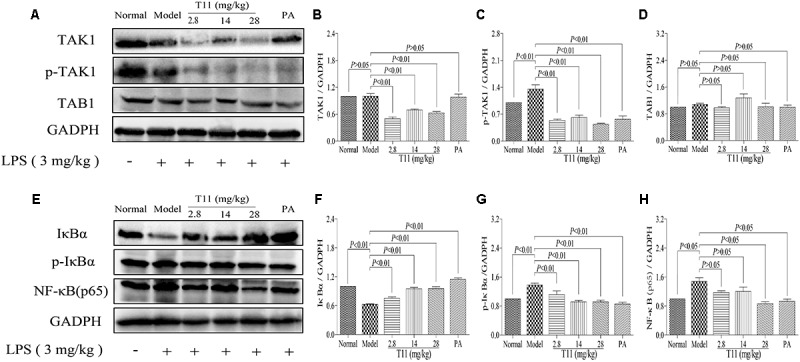
T11 could regulate NF-κB signaling pathway in LPS-induced liver injury in mice. **(A)** The proteins levels of TAK1, p-TAK1 and TAB1 were detected by western blot. **(B)** TAK1 expression. **(C)** p-TAK1 expression. **(D)** TAB1 expression. **(E)** The proteins levels of IκBα, p-IκBα and NF-κB (p65) were detected by western blot. **(F)** IκBα expression. **(G)** p-IκBα expression. **(H)** NF-κB (p65) expression. The data reveal as the mean ± SEM of 5–7 mice in each group. Group comparisons were performed by one-way analysis of variance (ANOVA) followed by *post hoc* Tukey’s test or Student’s *t*-test when appropriate. *P* < 0.05 was considered as statistical significance.

Given that T11 could effectively reduce LPS-induced cytokines (TNF-α, IL-6, and IL-1β) production in liver, which is responsible for the inhibition of NF-κB related signaling pathway, a key transcription factor of inflammation. The TAB1 molecule is a key regulator or adaptor protein in TAK1 phosphorylation processes and its down-stream inflammation cascade reaction, especially in NF-κB activation (Ajibade et al., 2012; Kobayashi et al., 2016). Thus, we investigated whether T11 could directly or indirectly block the activation of NF-κB signaling pathway. As shown in **Figures 10A–D**, LPS treatment could significantly up-regulate the expression of phosphorylation of TAK1, without affecting the expressions of TAK1 and TAB1. However, the expressions of TAK1 and p-TAK1 were significantly decreased after administration with T11 and PA, while the expression of TAB1 was slightly decreased. As shown in **Figures 10E–H**, LPS could significantly down-regulate the expression of IκBα and up-regulate the expressions of p-IκBα and NF-κB (p65). However, T11 and PA pretreatment could increase the expression of IκBα and dose-dependently inhibit the expressions of p-IκBα and NF-κB (p65). These results indicated that T11 could inhibit the activation of NF-κB signaling pathway and further reduce cytokines production.

### T11 Pre-treatment Regulated the Nrf2 Expression in LPS-Induced Mice

**FIGURE 11:**
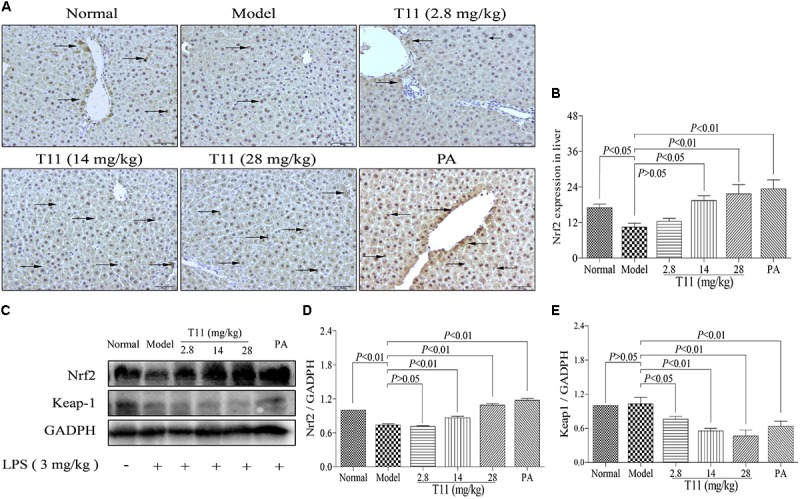
T11 could regulate Nrf2 signaling pathways in LPS-induced liver injury in mice. **(A)** Immunohistochemical staining of Nrf2 in liver tissues (magnification × 200, black arrows). **(B)** The expression of Nrf2 in liver. **(C)** The proteins levels of Nrf2 and keap1 were detected by western blot. **(D)** Nrf2 expression. **(E)** keap1 expression. The data reveal as the mean ± SEM of 5–7 mice in each group. Group comparisons were performed by one-way analysis of variance (ANOVA) followed by *post hoc* Tukey’s test or Student’s *t*-test when appropriate. *P* < 0.05 was considered as statistical significance.

In consideration of T11 could effectively reduce MAD level and increase SOD level in liver of ALI mice, which may be responsible for the activation of Nrf2 protein, a key regulator in oxidative stress and cytokines production in mice (Mahmoud et al., 2017). We detected Nrf2 and keap1 expression levels in liver tissues by IHC and western blot methods to further investigate the protective mechanisms of T11 on LPS-induced liver injury in mice. We found that the expression of Nrf2 was significantly decreased in model group (**Figures 11A,B**). By contrast, more expressions of Nrf2 were detected in the liver tissues from T11 pre-treated mice (**Figures 11A,B**). The result of western blot showed that the expression of Nrf2 was significantly decreased in model group, and the expression of keap1 was slightly decreased in model group (**Figures 11C–E**). Whereas, T11 and PA pre-treatment could dose-dependently up-regulate Nrf2 protein expression level and significantly down-regulate the expression of keap1 in liver tissues (**Figures 11C–E**). Therefore, T11 could activate Nrf2 signaling pathway to protect the liver from LPS-induced oxidative stress.

## Discussion

Accumulating evidences have shown that excessive oxidative stress and inflammatory responses play an essential role in the pathogenesis of ALI (Sukumaran et al., 2011). Many traditional Chinese herbal medicines have been have been used to treat liver diseases due to its unique pharmacological effects on anti-inflammatory and anti-oxidant (Ding et al., 2012; Lam et al., 2016). T11 is a diterpene component extracted from TwHF, a traditional Chinese herb. These components have extensive pharmacological effects (Lee et al., 2007). However, up to now, the pharmacology activities of T11 has not been well investigated. Therefore, in the present study, we investigated whether T11 could inhibit inflammatory responses and oxidative stress *in vitro* and *in vivo* for the first time, and further explored its therapeutic effect on ALI.

To evaluate the anti-inflammation and anti-oxidant properties of T11 *in vitro*, Lipopolysaccharide (LPS) -induced RAW264.7 cells inflammation and oxidation damage model was applied in our study. Macrophage stimulated with LPS could increase the release of pro-inflammatory cytokines, such as TNF-α, IL-1β, and IL-6 (Chen et al., 2017). In addition, LPS can also stimulate the over production of ROS and nitric oxide (NO), which aggravates oxidative stress in RAW264.7 cells (Jung et al., 2014). Interestingly, our results showed that T11 treatment could dose-dependently inhibit the secretion of pro-inflammation cytokines (i.e., TNF-α, IL-1β and IL-6) in LPS-stimulated RAW264.7 cells. Meanwhile, we also found that T11 significantly suppressed the generations of ROS and NO in LPS-stimulated RAW264.7 cells. These results demonstrated that the anti-inflammatory and anti-oxidant activities of T11.

Previous data have shown that T11 could effectively inhibit the release of inflammatory mediators and ROS to exert the effects of anti-inflammatory and anti-oxidant *in vitro*. More and more evidences have shown that LPS stimulated cells by binding to and activating toll-like receptor 4 (TLR4), and then primarily activates the inflammatory pathways, such as TAK-1/NF-κB signal pathways (Kumar et al., 2017; Nepali et al., 2017). Meanwhile, activated pro-inflammatory pathways, including TAK-1/NF-κB signal pathways, can further promote the secretion of pro-inflammation cytokines (i.e., TNF-α, IL-1β, and IL-6) and aggravate inflammatory injury (Wang et al., 2014). Based on this, we speculated that the anti-inflammatory property of T11 may be achieved via inhibiting the activation and transcription of TAK-1/NF-κB signal pathways. Our results demonstrated that T11 pre-treatment could markedly inhibit the expressions of TAK-1, p-TAK-1, p-IκBα and NF-κB (p65) in LPS-stimulated RAW264.7 cells. The results of molecular docking also revealed that T11 had a good binding affinity with TAK1-TAB1 protein and the affinity is similar to the known TAK1-TAB1 protein inhibitor. The data suggested that T11 suppressed TAK-1/NF-κB signal pathways to exert the anti-inflammatory effect in LPS-induced RAW 264.7 cells.

In addition, overproduction of ROS is closely related to oxidative stress in LPS-induced RAW 264.7 cells (Sheu et al., 2018). Nrf2 is a regulator of ROS, which could regulate ROS homeostasis in wide variety of cell types (Zhai et al., 2012). T11 could significantly inhibit the secretion of ROS in LPS-stimulated RAW264.7 cells. These facts may activate Nrf2 signaling pathway to exert anti-oxidation. The results showed that T11 dose-dependently activated the expression of Nrf2. Meanwhile, the results of molecular docking revealed that the affinity between T11 and Keap1 protein is similar to di-acetamides, which are known as non-electrophilic activators of Nrf2 (Jain et al., 2015). In addition, the dual-luciferase reporter gene also evidenced that T11 largely enhanced the transcription of ARE-dependent luciferase gene, suggesting that T11 could exert anti-oxidation by activating Nrf2 signaling pathway.

In clinical, accumulated evidences have shown that the pathogenesis of ALI is closely related to inflammation and oxidative stress in patients (Li et al., 2016). Meanwhile, many drugs with the properties of anti-inflammation and anti-oxidant, have been used to relieve ALI (Li et al., 2016). Previous studies have evidenced that T11 has the effects of anti-inflammation and anti-oxidant on LPS-stimulated RAW264.7 cells. LPS shows a notable oxidative stress and inflammatory responses properties in animals by decreasing plasma antioxidant capacity and activating the TAK1/NF-κB signal pathways to aggravate the liver injury (Ivshina et al., 2015). On this Based, we further investigated that whether T11 could treat ALI induced by LPS in mice. In the present study, the results suggested that T11 could ameliorate the inflammatory response (i.e., neutrephil and macrophages infiltration) in liver of LPS-induced ALI model. The serum and liver levels of ALT and AST were significantly decreased after pre-treatment with T11. In addition, we also found that treatment with T11 without LPS did not cause liver damage in mice. These results indicated that T11 may be a active compound to treat ALI.

LPS-induced ALI involves the inflammatory response of liver (Wang et al., 2014). Inflammatory cells, such as macrophages and neutrophils, were activated and further release massive pro-inflammatory cytokines to aggravate liver injury (Ivshina et al., 2015). Interestingly, pre-treatment with T11 significantly inhibited the activation of macrophages and neutrophils in liver of LPS-induced ALI model. Meanwhile, T11 also markedly reduced the secretion of pro-inflammatory cytokines in the liver. The overproduction of pro-inflammatory cytokines is closely related to the activation of inflammatory signaling pathways, including TAK-1 signaling pathways and NF-κB signal pathways (Nepali et al., 2017). *In vitro* study, we have shown that T11 exerts the anti-inflammatory effect by inhibiting the expressions of TAK-1/NF-κB signal pathways. Further study also evidenced that T11 could down-regulate the expressions of TAK-1, p-TAK-1, p-IκBα and NF-κB (p65) in liver. These results certified that suppression of TAK-1/NF-κB signal pathways may be the potential mechanism of T11 against LPS-induced ALI.

Oxidative stress also can increase the expressions of pro-inflammatory genes, which involves in the occurrence and development of many diseases, including ALI (Kamouni et al., 2017). As a nuclear factor-E2-related factor 2, Nrf2 can relieve LPS-induced ALI in mice by suppressing inflammation and oxidative stress (Keleku-Lukwete et al., 2017). In our study, pre-treatment with T11 could markedly increase the activity of SOD and reduce the level of MDA in LPS-induced ALI model. The results implied that T11 could inhibit oxidative stress in LPS-induced ALI mice. Moreover, the expression of liver Nrf2 protein was significantly up-regulated after pre-treatment with T11 in LPS-induced ALI. These results are in agreement with the *vitro* results. Therefore, the protective effect of T11 on LPS-induced ALI was achieved by activating Nrf2 signaling pathway.

## Conclusion

Our findings firstly demonstrated that T11 possess anti-inflammatory and anti-oxidant effect on LPS-stimulated RAW 264.7 cells. Its mechanism manifested as inhibiting the activation of TAK-1/NF-κB signaling pathways and up-regulating Nrf2 expression (**Supplementary Figure S7**). Subsequently, we also found that T11 could protect against LPS-induced ALI by suppressing inflammatory response and oxidative stress, which provide beneficial evidences for the application of T11 in the prevention of inflammation and oxidative stress-associated diseases, especially ALI.

## Author Contributions

Y-QY, X-TY, KW, Z-YL, R-MT, and L-LW participated in the experiments. H-TX, Y-SW, X-SL, and WM contributed to the data analysis and study analysis. PX and BL designed the experiment. All authors read and approved the final manuscript.

## Conflict of Interest Statement

The authors declare that the research was conducted in the absence of any commercial or financial relationships that could be construed as a potential conflict of interest.

## Abbreviations

ALI: acute liver injury
ALT: alanine amiotransferase
AST: aspartate aminotransferase
IL-1β: Interleukin-1β
IL-6: Interleukin-6
LPS: lipopolysaccharides
MDA: malondialdehyde
Nrf2: Nuclear Factor Erythroid 2 Related Factor 2
ROS: reactive oxygen species
SOD: superoxide dismutase
T11: triptriolide
T9: triptolide
TNF-α: tumor necrosis factor-alpha
TwHF: *Tripterygium wilfordii* Hook F.
