# Supplementary material for: Triptriolide Alleviates Lipopolysaccharide-Induced Liver Injury by Nrf2 and NF-κB Signaling Pathways
Source: Front Pharmacol. 2018 Aug 29;9:999. doi: 10.3389/fphar.2018.00999 (PMC6124152; doi:10.3389/fphar.2018.00999)
Supplement: Supplementary file 1 [file Data_Sheet_1.doc]

**Triptriolide Alleviates Lipopolysaccharide-Induced Liver Injury by Nrf2 and NF-κB Signaling Pathways**

***Yi-Qi Yang^1ǂ^, Xiao-Teng Yan^2ǂ^, Kai Wang^1^, Rui-Min Tian^3^, Zhao-Yu Lu^3^, Li-Lan Wu^3^, Hong-Tao Xu^4^, Yun-Shan Wu^1,3^, Xu-Sheng Liu^1,3^, Wei Mao^1,3^, Peng Xu^1,3^,*, Bo Liu^1,3^,****

**Part I: Acute toxicity study of TA (detoxification extract of TwHF) and TB (extract of TwHF) in mice**

1. **Animals**

7-8 weeks old KM mice of both sexes (20 ± 2 g) were obtained from the Laboratory Animal Services Center, Guangzhou University of Chinese Medicine (Guangzhou, China). Mice were housed and maintained under specific pathogen-free conditions where they received standard food and sterilized water ad libitum under constant temperature (20-25℃) and humidity (65-70%) with a 12 light/dark cycle. Experimental protocols were approved by the Animal Ethics Committee of Guangzhou University of Chinese Medicine.

**2. Acute oral toxicity**

Acute oral toxicity study was performed according the method by Li [Li et al., 2014], with slight modification. Briefly, the mice were fasted for 12 h before administration of the TA and TB, and assigned to groups using computerized random sort program so that the body weight means for every group as follows: 130 KM mice were allotted randomly in 13 groups of 10 mice each (five males and five females). The control group received sterile water (0.1ml/10 g, p.o.). TA was administered to mice at graded doses of 760, 920, 1160, 1400, 1800 and 2400 mg/kg. TB was administered to mice at graded doses of 280, 360, 480, 600, 720 and 860 mg/kg. The toxic symptoms, such as sedation, alertness, spontaneous motor, unhairing, anxiety, were observed continuously for 3 h after TA and TB treatment. All mice were further observed twice a day up to 14 days for behavior changes and mortality (**Table S1**).

Table S1. Acute toxicity study of TA and TB in mice

| Dose（mg/kg） | | Animals | |  | Effects |
| --- | --- | --- | --- | --- | --- |
|  |  | Dead/ Total number of animals | Mortality (%) |  | Toxic symptom |
| Control | 0 | 0 | 0 | None | |
| TA | 760 | 0 | 0 | None | |
|  | 920 | 0 | 0 | None | |
|  | 1160 | 0 | 0 | None | |
|  | 1400 | 0 | 0 | None | |
|  | 1800 | 0 | 0 | None | |
|  | 2400 | 1 | 0.1 | Yes | |
| TB | 280 | 0 | 0 | None | |
|  | 360 | 5 | 0.5 | Yes | |
|  | 480 | 4 | 0.4 | Yes | |
|  | 600 | 4 | 0.4 | Yes | |
|  | 720 | 7 | 0.7 | Yes | |
|  | 860 | 9 | 0.9 | Yes | |

**References:**

[Li, Z](http://nc.yuntsg.com/pubmed/?term=Li Z[Author]&cauthor=true&cauthor_uid=24486111)., [Qiao, Y](http://nc.yuntsg.com/pubmed/?term=Qiao Y[Author]&cauthor=true&cauthor_uid=24486111)., [Li, J](http://nc.yuntsg.com/pubmed/?term=Li J[Author]&cauthor=true&cauthor_uid=24486111)., [An, C](http://nc.yuntsg.com/pubmed/?term=An C[Author]&cauthor=true&cauthor_uid=24486111)., [Hu, K](http://nc.yuntsg.com/pubmed/?term=Hu K[Author]&cauthor=true&cauthor_uid=24486111)., [Tang, M](http://nc.yuntsg.com/pubmed/?term=Tang M[Author]&cauthor=true&cauthor_uid=24486111). (2014). Acute and sub-chronic toxicity studies of the extract of Thunberg Fritillary Bulb. [*Regul Toxicol Pharmacol*.](http://nc.yuntsg.com/one1.do) 68, 370-377. doi: 10.1016/j.yrtph.2014.01.007.

**Part Ⅱ: The effect of treatment with T11 without injection LPS on liver**

1. **Animals**

Male BALB/c mice (20 ± 2 g) were obtained from the Laboratory Animal Services Center, Guangzhou University of Chinese Medicine (Guangzhou, China). Mice were housed and maintained under specific pathogen-free conditions where they received standard food and sterilized water ad libitum under constant temperature (20-25℃) and humidity (65-70%) with a 12 light/dark cycle. Experimental protocols were approved by the Animal Ethics Committee of Guangzhou University of Chinese Medicine.

1. **Treatment**

Mice were randomly divided into 4 groups (n = 6 per group): 1) Normal (0.4 % CMC), 2) T11-L (2.8 mg/kg, Low dose), 3) T11-M (14 mg/kg, Middle dose), 4) T11-H (28 mg/kg, High dose). Normal control group was given 0.4 % CMC (10 ml/kg, p.o.) once daily, while T11 groups were administrated with 2.8, 14 and 28 mg/kg T11 (10 ml/kg, p.o.) once daily. After 7 days treatment with T11, all mice were killed humanely to evaluate the effect of T11 on liver. The serum samples and liver tissues were isolated for downstream biochemistry and histopathology analysis. The Serum Biochemical index, including ALT and AST, was assayed at clinical lab of Guangdong province hospital of traditional Chinese medicine.

1. **Statistical Analysis**

Statistical analysis was performed with SPSS version 18.0 (Chicago, IL, USA). All results were expressed as mean ± SEM. Group comparisons were performed by one-way analysis of variance (ANOVA) followed by post hoc Tukey’s test or Student’s t-test when appropriate. *P* < 0.05 was considered as statistically signiﬁcant.

1. **Results**

3.1 Serum Biochemical analysis

As shown in **Figure S1**, treatment with T11 without LPS injection did not increase the levels of ALT and AST in serum. These data showed that T11 did not cause liver injury in normal mice.


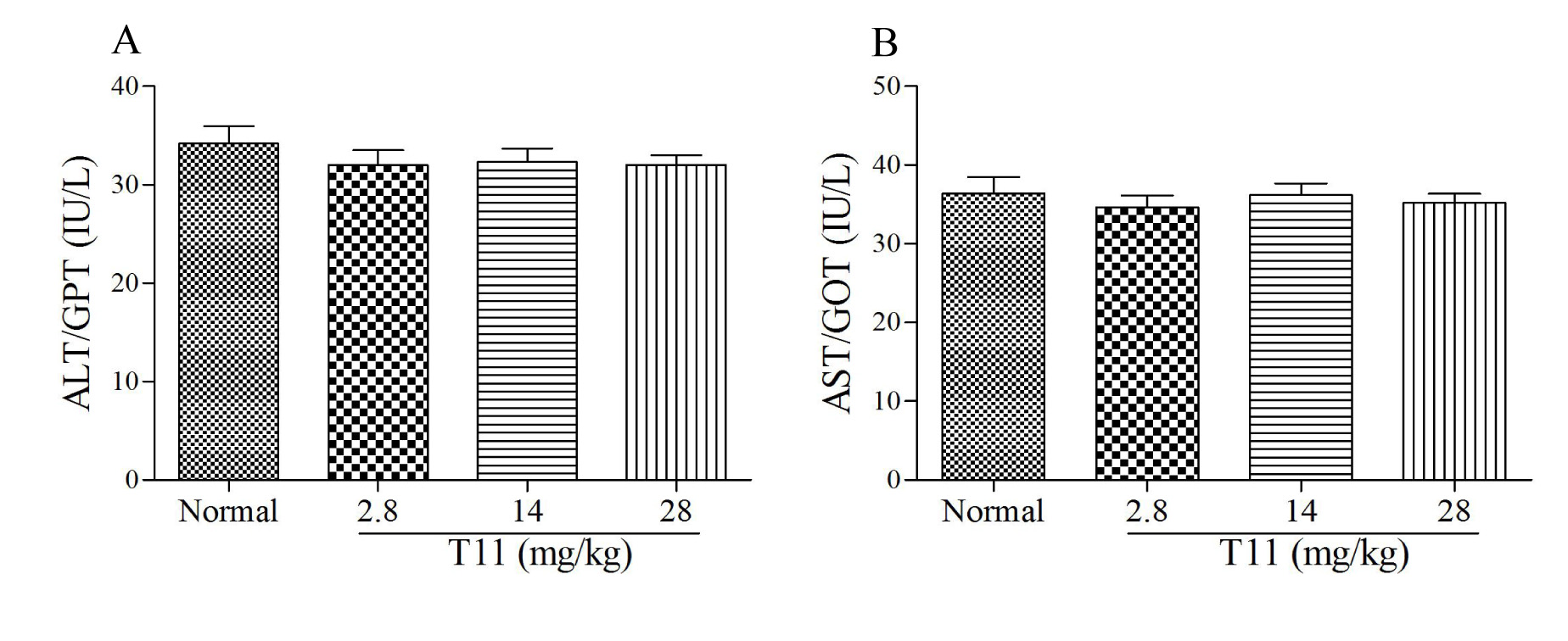


**FIGURE S1** The effect of treatment with T11 without LPS injection on liver. (A) Effect T11 on ALT level in serum. (B) Effect T11 on AST level in serum. The data reveal as the mean±S.E.M of 5 mice in each group. Group comparisons were performed by one-way analysis of variance (ANOVA) followed by post hoc Tukey’s test or Student’s t-test when appropriate. ^*^*P* < 0.05 compared with Normal group.

3.2 Liver histopathology analysis

As shown in Figure S2, treatment with T11 without LPS injection did not cause obvious change for the appearance characteristics of liver images in mice (Figure S2A). In addition, the result of liver histopathology also showed that T11 treatment did not damage the construction of liver and did not increase the inflammatory cell infiltration in liver.


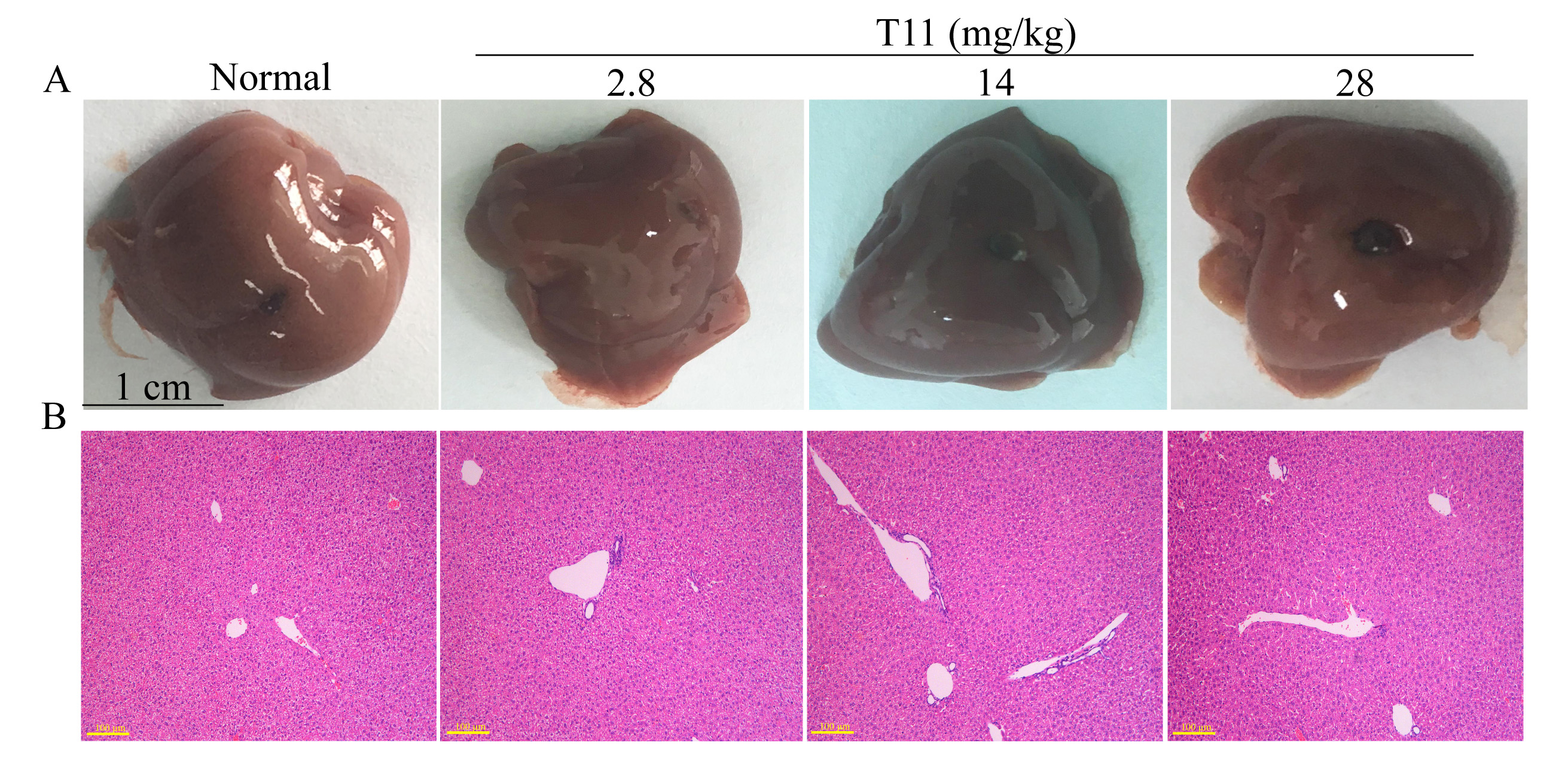


**FIGURE S2** The effect of treatment with T11 without LPS injection on liver of mice. (A) Representative liver pictures of mice in each group. (B) Representive images of hematoxylin and eosin (H&E) staining in liver (magnification ×100).

**Part III: The effect of T11 on LPS-induced depression-like behaviors and liver fibrotic changes**

1. **Animals**

Male BALB/c mice (18-20 g) were used for this experiment. The BALB/c mice were purchased from Laboratory Animal Services Center, Guangzhou University of Chinese Medicine (Guangzhou, China). After arrival in our facility, the National Institutes of Health Guide for the use of Laboratory animals were performed to raise all animals. Animals were raised in a standard environment condition with the temperature at 22 ± 2 ℃ under a 12 h dark/light cycle, and allowed free access to sterilized water and standard food. Experimental protocols were reviewed and approved by Animal Care and Use Committee (Guangzhou university of Chinese medicine, Guangzhou).

ALI mice model was induced as described by Wang . Briefly, mice were randomly divided into 6 groups (n = 6 per group): 1) Normal (0.4 % CMC), 2) Model (3 mg/kg LPS), 3) LPS+T11-L (2.8 mg/kg, Low dose), 4) LPS+T11-M (14 mg/kg, Middle dose), 5) LPS+T11-H (28 mg/kg, High dose), 6) LPS+PA (Prednisone acetate, PA as a positive control, 1.3 mg/kg ). T11 and PA were pretreated by gavage for 7 days. LPS was administered through intraperitoneal injection at 6 h before sacrificed. LPS-induced depression-like behaviors was recorded by camera (Nikon, Japan) and liver tissues were isolated to evaluate liver fibrotic changes using sirius red staining (Nakles et al ., 2011).

1. **Results**
   1. The effect of T11 on LPS-induced depression-like behaviors

Evidences have shown that LPS can induce the depression-like behaviors in mice (Adebesin et al., 2017). We found that treatment with T11 could significantly improve LPS-induced depression-like behaviors in mice, such as curled up and inactive (**Figure S3A**). Meanwhile, LPS could obviously increase the secretions in eyes. However, treatment with T11 significantly inhibited eye secretions increase (**Figure S3B**). These results showed that T11 could protect the body from LPS induced injury in mice.


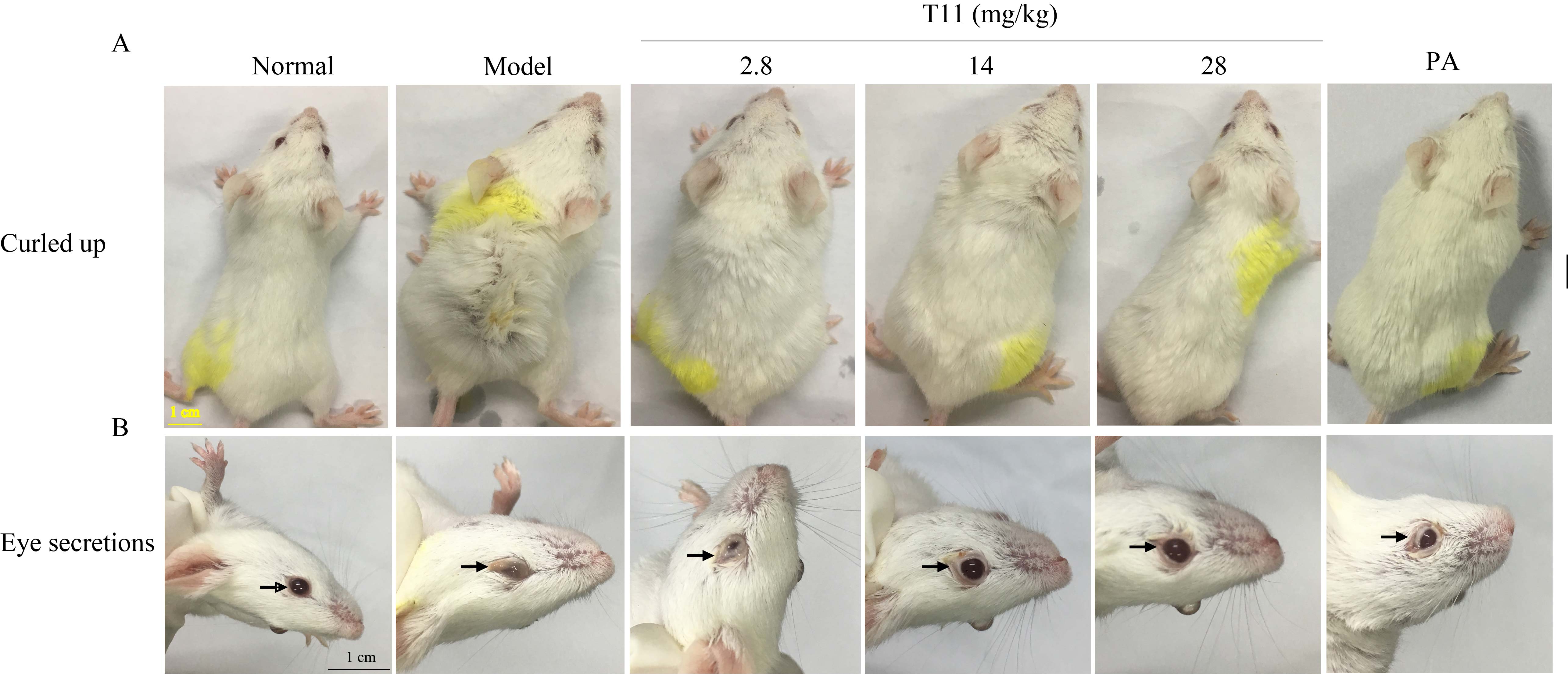


**FIGURE S3** The effect of T11 on LPS-induced depression-like behaviors. (A) T11 improved the depression-like behaviors in LPS-induced mice. (B) T11 reduced eye secretions in LPS-induced mice.

- 1. Sirius red staining

As shown in Figure S4, we found that injection with LPS in mice for 6 h can not cause obvious collagen fibers production in liver as compared to normal group. Meanwhile, the intensities of collagen fibers also have no significant change after treatment with T11 as compared to model group (**Figure S4**).


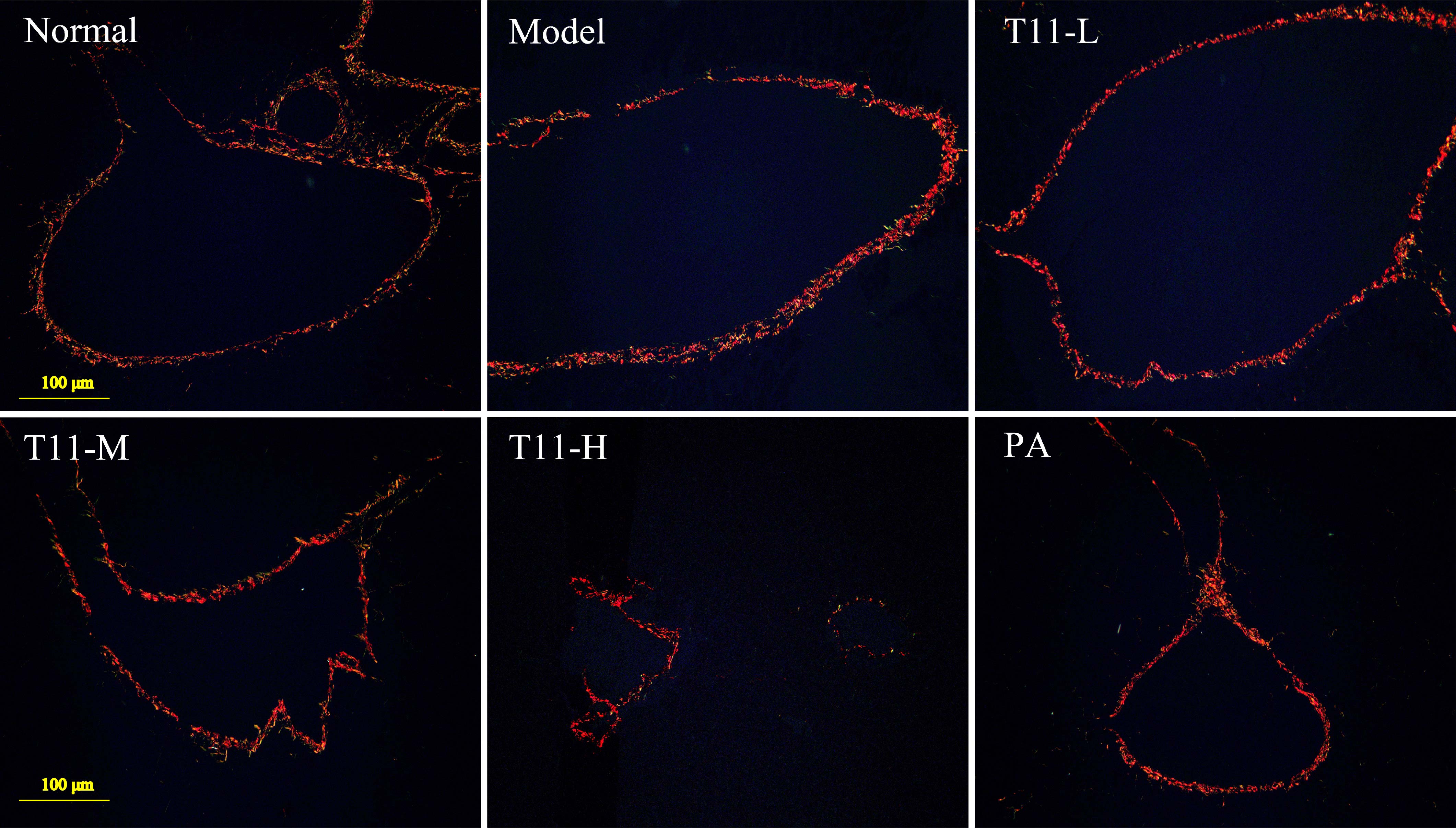


**FIGURE S4** The effect of T11 on the intensity of collagen fibers in LPS-induced ALI mice. (A) Sirius red staining (original magnification, ×100; scale bar=100 µm).

**References:**

Wang, W., Wu, L., Li, Q., Zhang, Z., Xu, L., Lin, C., et al. (2018). Madecassoside prevents acute liver failure in LPS/D-GalN-induced mice by inhibiting p38/NF-κB and activating Nrf2/HO-1 signaling. 103, 1137-1145. doi: 10.1016/j.biopha.2018.04.162.

[Adebesin, A](http://nc.yuntsg.com/pubmed/?term=Adebesin A[Author]&cauthor=true&cauthor_uid=28647678)., [Adeoluwa, O. A](http://nc.yuntsg.com/pubmed/?term=Adeoluwa OA[Author]&cauthor=true&cauthor_uid=28647678)., [Eduviere, A. T](http://nc.yuntsg.com/pubmed/?term=Eduviere AT[Author]&cauthor=true&cauthor_uid=28647678)., [Umukoro, S](http://nc.yuntsg.com/pubmed/?term=Umukoro S[Author]&cauthor=true&cauthor_uid=28647678). (2017). Methyl jasmonate attenuated lipopolysaccharide-induced depressive-like behaviour in mice. [J Psychiatr Res.](http://nc.yuntsg.com/one1.do) 94, 29-35. doi: 10.1016/j.jpsychires.2017.06.007.

Nakles, R. E., Shiffert, M. T., Díaz-Cruz, E. S., Cabrera, M. C., Alotaiby, M, Miermont, A. M., et al. (2011). Altered AIB1 or AIB1Δ3 expression impacts ERα effects on mammary gland stromal and epithelial content. [Mol Endocrinol.](http://nc.yuntsg.com/show.do?q=21292825&my=1531535822318) 25, 549-563. doi: 10.1210/me.2010-0114.

**Part Ⅳ: Molecular Docking Analyses of Triptriolide and TAK1-TAB1 protein or Keap1 protein**

1 The method of Molecular Docking

In molecular docking, the silico analyses were performed to illuminate the major binding mode, and the binding affinity of Triptriolide constituents with the target receptor, such as TAK1-TAB1 protein (PDB code: 5J9L) **(Tan et al., 2017)** or Keap1 protein (PDB code: 4XMB) **(Jain et al., 2015)**. AutoDock Tool was applied to analyze the space of all the binding sites of ligand and receptor. The volume and position of grid box were extracted according to the bind pocked. The size of grid box was designed, which is suited for encompassing the ligand structure and the possible binding residues of the receptor. The AutoDock Vina was applied for screening. The docking score for the binding affinity of each ligand and receptor were obtained by AutoDock Vina. In addition, the relationship between score and binding were directly analyzed by PyMOL (PyMOL Molecular Graphics System, USA).

2 Results

2.1 Molecular Docking Analyses of Triptriolide and TAK1-TAB1 protein

As shown in **Figure S5**, the structure of human TAK1-TAB1 protein (PDB code: 5J9L) was selected for analyzing molecular docking. Triptriolide had a good binding affinity for the protein model (**Figure S5A**). In addition, a carbonyl group of Triptriolide formed hydrogen bonds with Val 42, Ser 111, and Leu 163 of human TAK1-TAB1 protein (**Figure S5B**). Further studies will be performed to explore the relationship between Triptriolide and TAK1-TAB1 protein. The information of benchmark is obtained by Tan **(Tan et al., 2017)**. The benchmark, N-(4-((2-((4-(4-methylpiperazin-1-yl)phenyl)amino)-7H-pyrrolo[2,3-d]pyrimidin-4-yl)oxy)phenyl)acrylamide, is the inhibitor for TAK1-TAB1 protein (PDB code: 5J9L) **(Tan et al., 2017)**.


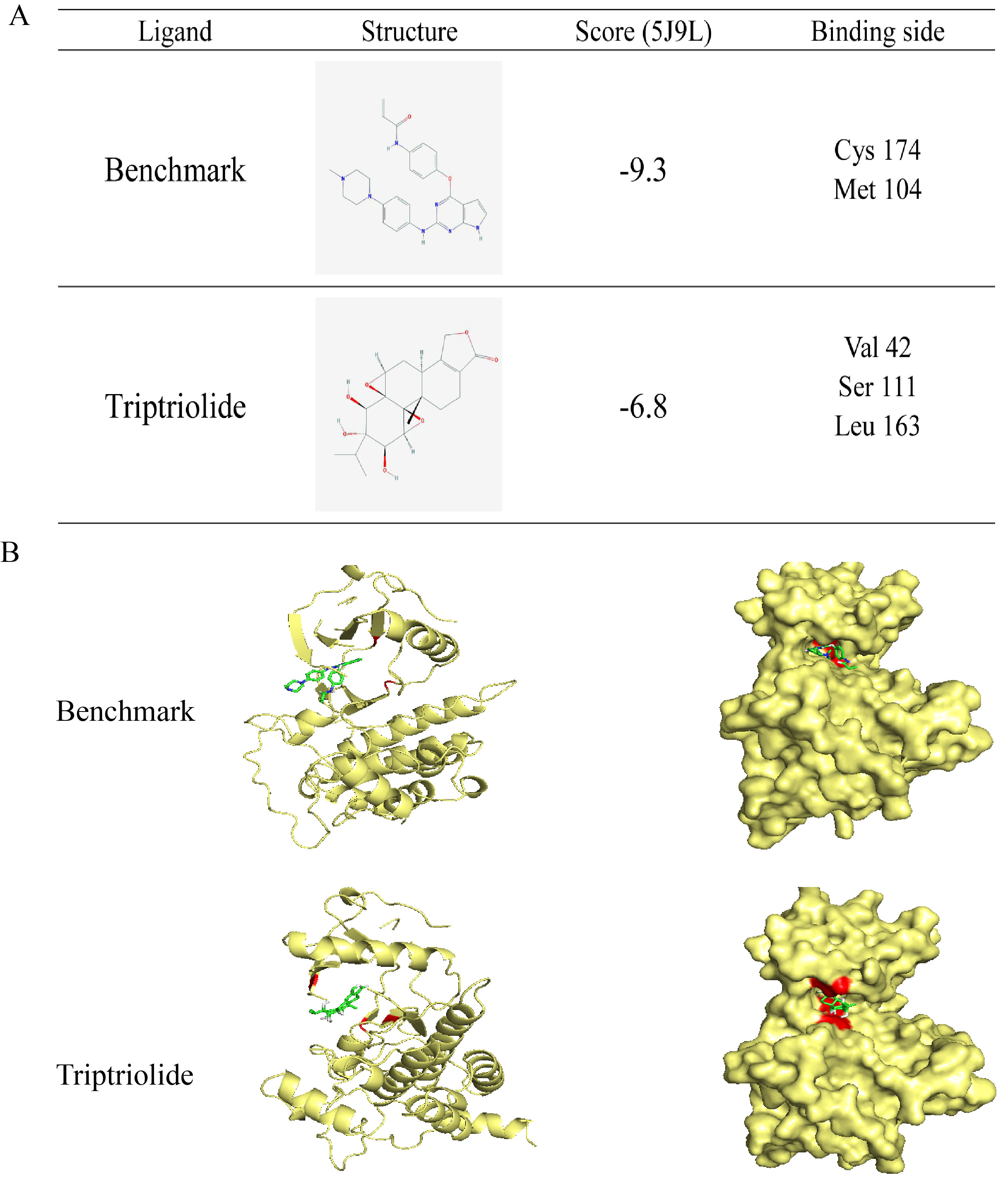


**FIGURE S5.** The molecular docking results of Triptriolide and TAK1-TAB1 protein. (A) The favorable binding positions of Triptriolide with lowest binding free energy. (B) Molecular docking analysis illustrates the favorable binding positions of Triptriolide with lowest binding free energy in the binding site of human TAK1-TAB1 protein.

2.2 Molecular Docking Analyses of Triptriolide and Keap1 protein

As shown in **Figure S6**, the structure of human Keap1 (PDB code: 4XMB) was selected for analyzing molecular docking. Triptriolide had good binding affinity with the protein model (**Figure S6A**). In addition, a carbonyl group of Triptriolide formed hydrogen bonds with Agr 483, Ser 555, Tyr 572 and Ser 602 of human Keap1 protein (**Figure S6B**). Further studies will be performed to explore the relationship between Triptriolide and Keap1 protein. The benchmark is 2,2’-(naphthalene-1,4-diylbis (((4-methoxyphenyl)sulfonyl) azanediyl)) diacetamide, which is the agonist for Keap1 (PDB code: 4XMB) **(Jain et al., 2015)**.


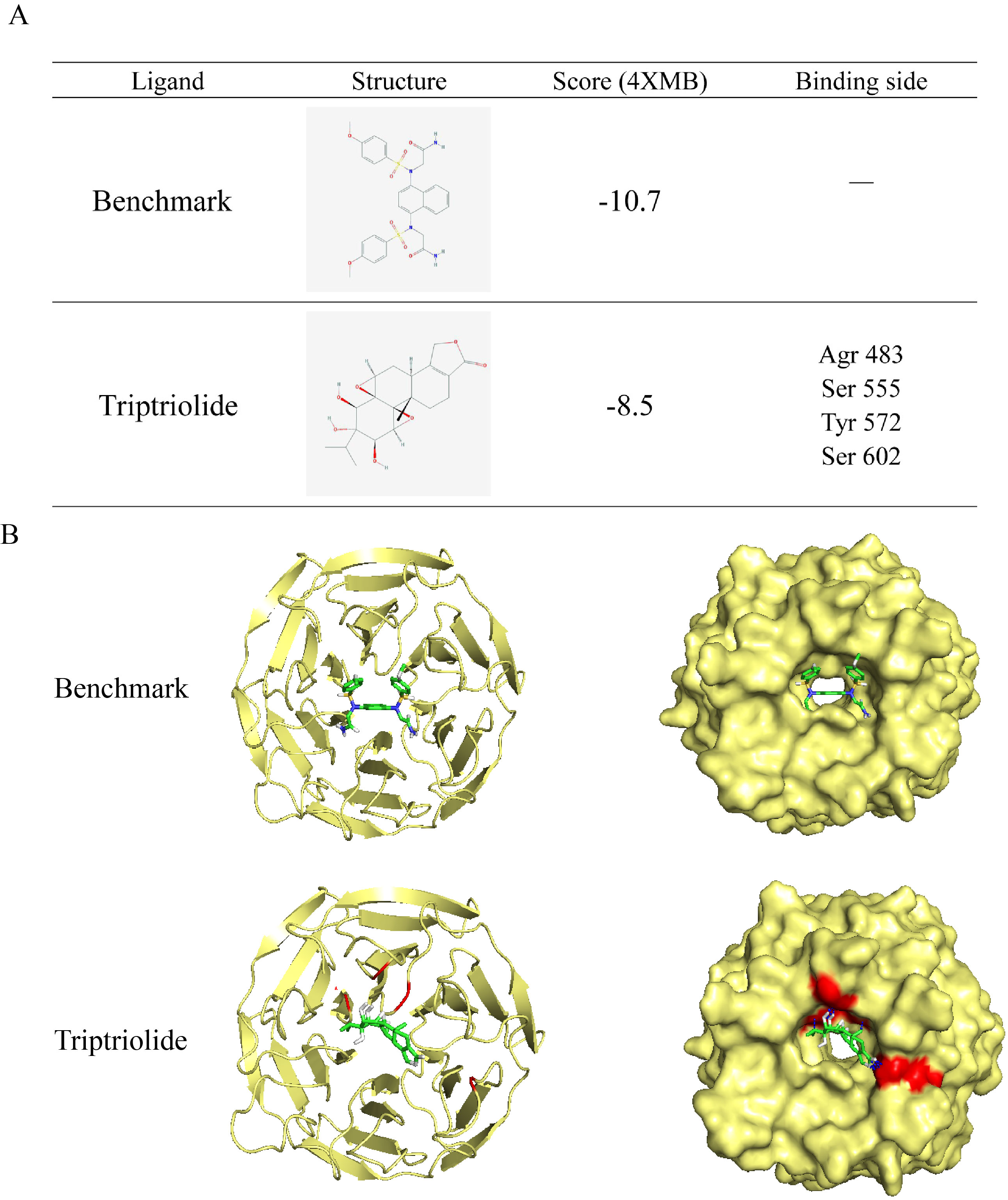


**FIGURE S6.** The molecular docking results of Triptriolide and human Keap1 protein. (A) The favorable binding positions of Triptriolide with lowest binding free energy. (B) Molecular docking analysis illustrates the favorable binding positions of Triptriolide with lowest binding free energy in the binding site of human Keap1 protein.

**References**

Tan, L., Gurbani, D., Weisberg, E. L., Hunter, J. C., Li, L., et al., (2017). Structure-guided development of covalent TAK1 inhibitors. [*Bioorg Med Chem*](http://nc.yuntsg.com/one1.do). 25 (3), 838-846. doi: 10.1016/j.bmc.2016.11.035.

Jain, A. D., Potteti, H., Richardson, B. G., Kingsley, L., Luciano, J. P., et al., (2015). Probing the structural requirements of non-electrophilic naphthalene-based Nrf2 activators. *Eur J Med Chem*. 103, 252-268. doi: 10.1016/j.ejmech.2015.08.049.

**Graphical abstract**


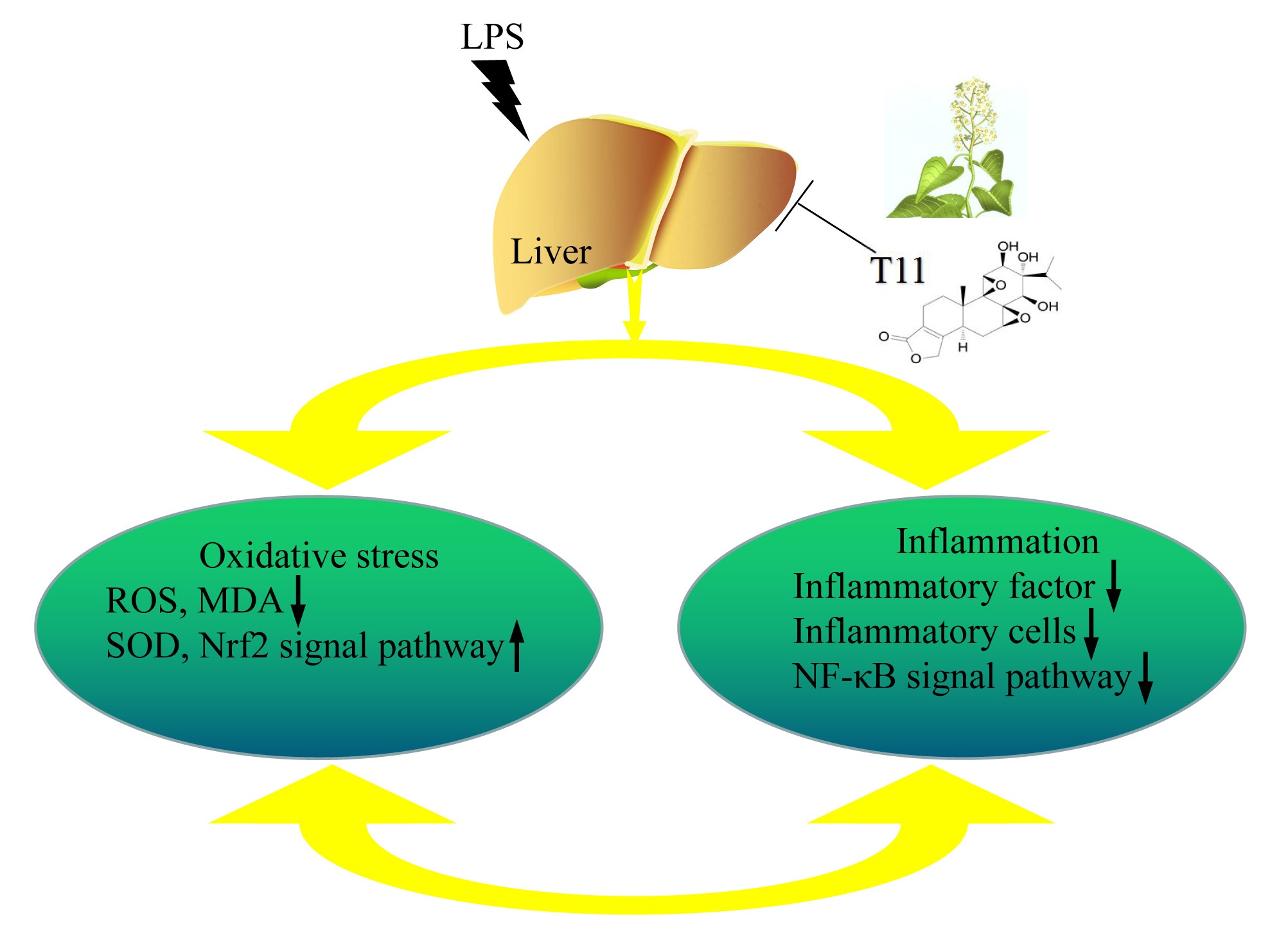


**FIGURE S7. Triptriolide Alleviates Lipopolysaccharide-Induced Liver Injury by Nrf2 and NF-kB Signaling Pathways.**
